# Accuracy of tibial positioning in the frontal plane: a prospective study comparing conventional and innovative techniques in total knee arthroplasty

**DOI:** 10.1007/s00402-020-03389-4

**Published:** 2020-03-02

**Authors:** R. K. Zahn, F. Graef, J. L. Conrad, L. Renner, C. Perka, H. Hommel

**Affiliations:** 1 Center for Musculoskeletal Surgery, Charité–University Medicine Berlin, Charitéplatz 1, 10115 Berlin, Germany; 2 Berlin Institute of Health, Anna-Louisa-Karsch-Straße 2, 10178 Berlin, Germany; 3 Department of Orthopaedics, Märkisch-Oderland Hospital, Brandenburg Medical School Theodor Fontane, Wriezen, Germany

**Keywords:** Total knee arthroplasty, Tibial positioning, Mechanical axis, Extramedullary, Intramedullary, Navigation, PSI

## Abstract

**Background:**

Coronal alignment of the tibial component determines functional outcome and survival in total knee arthroplasty (TKA). Innovative techniques for tibial instrumentation have been developed to improve accuracy and reduce the rate of outliers.

**Methods:**

In a prospective study, 300 patients were allocated to four different groups using a randomization process (two innovative and two conventional) techniques of tibial instrumentation (conventional: extramedullary, intramedullary; innovative: navigation and patient-specific instrumentation (PSI); *n* = 75 for each group). The aims were to reconstruct the medial proximal tibial angle (MPTA) to 90° and the mechanical tibio-femoral axis (mTFA) to 0°. Both angles were evaluated and compared between all groups three months after the surgery. Patients who presented with a postoperative mTFA > 3° were classified as outliers.

**Results:**

The navigation and intramedullary technique both demonstrated that they were significantly more precise in reconstructing a neutral mTFA and MPTA compared to the other two techniques. The odd’s ratio (OR) for producing outliers was highest for the PSI method (PSI OR = 5.5, *p* < 0.05; extramedullary positioning OR = 3.7, *p* > 0.05; intramedullary positioning OR = 1.7, *p* > 0.05; navigation OR = 0.04, *p* < 0.05). We could only observe significant differences between pre- and postoperative MPTA in the navigation and intramedullary group. The MPTA showed a significant negative correlation with the mTFA in all groups preoperatively and in the extramedullary, intramedullary and PSI postoperatively.

**Conclusion:**

The navigation and intramedullary instrumentation provided the precise positioning of the tibial component. Outliers were most common within the PSI and extramedullary technique. Optimal alignment is dependent on the technique of tibial instrumentation and tibial component positioning determines the accuracy in TKA since mTFA correlated with MPTA pre- and postoperatively.

## Introduction

Coronal alignment of the tibial and femoral components determines the functional outcome and survival after TKA [1, 2]. The mechanical tibiofemoral axis (mTFA) is considered to be aligned physiologically if the axis is within a range of ± 3° [3]. A mTFA > 3° post-TKA is defined as an outlier. Tibial malalignment is associated with an impaired function, increased complication risk and reduced survival rates [4, 5]. Intra- or extramedullary tibial instrumentation are conventional techniques and have likewise been demonstrated to achieve good outcomes. Nevertheless, high rates of outliers of up to 37% have been published [6].

Extramedullary instrumentation is most frequently used due to its easy handling as well as decreased invasiveness and morbidity compared to the intramedullary technique, which is of limited use in excessive tibial deformities [7, 8]. Innovative methods such as computer-assisted navigation or individualized cutting blocks in PSI have been developed to increase the accuracy of component positioning, reduce the rate of outliers and improve the function and survival rates in TKA [9, 10].

To our knowledge, results of tibial component positioning in the frontal plane in intra- and extramedullary positioning as well as navigation and PSI have not been directly compared, yet. Therefore, the purpose of this study was to investigate the accuracy of these four different tibial positioning techniques and emphasize the specific characteristics of conventional and innovative techniques.

## Methods

### Study design

In this prospective study, 300 consecutive TKAs in 300 patients with primary osteoarthritis (123 men, 177 women, age: 67 ± 7.5 years; body mass index (BMI): 29. 2 ± 2.8 kg/m^2^) were included between 2012–2015. Exclusion criteria were: preoperative extension deficit > 20°, valgus or varus malalignment > 15° and previous surgery of the affected joint. Patients deemed eligible for study inclusion were asked to participate in the study. Written informed consent was obtained from all patients. The local ethics committee approved this study (Landesärztekammer Brandenburg, approval number: S 12 (a) 2012).

Sample size calculations were performed using nQuery (Statistical Solutions Ltd, Cork, Ireland). Group sizes were determined given a significance level of 0.05, number of groups = 4, a power of 80% and a common standard deviation of the MPTA as stated in the literature for the gap-balancing technique of SD = 2.0 [11]. Given this, each group consisted of *n* = 75 patients. For randomization, a consecutively numbered list with 300 positions was created. Each number was randomly assigned one operative technique using a spreadsheet (Excel, Microsoft Corporation, New Mexico, USA). Subsequently, patients were allocated to one of the four operative techniques in ascending order of their operation date in the clinic.

Preoperatively, the implant components were digitally planned perpendicular to the mechanical femoral and tibial axes using x-rays and the software mediCAD (Hectec GmbH, Altdorf, Germany). In all preoperative plannings, both the mTFA and MPTA were aimed to be reconstructed to their physiological angle of 0° and 90°, respectively.

### Operative technique

All operations were performed by one single senior surgeon (H.H.). A medial parapatellar approach was used and surgery took place under general anesthesia. All TKAs were operated using the gap-balancing and tibia-first technique [12]. Patients with navigated TKA or extramedullary instrumentation were implanted a TC PLUS PRIMARY (Smith & Nephew, Memphis, TN, USA). In PSI and intramedullary the Journey II CR (Smith & Nephew, Memphis, TN, USA) was used.

In extramedullary instrumentation, the cutting jig was positioned with the orientation to the anteromedial tibial crest, the center of the upper ankle and the second metatarsal bone [13]. When the intramedullary instrumentation was used, the rod of the instrumentation system was entered through an entry point located at the center of the tibial plateau. The rod was inserted at least 20 cm into the tibial diaphysis [14]. Computer-assisted navigation was conducted using the PI Galileo system, which works via landmarks that are defined through infrared optical markers [15]. In the PSI group, the Visionare Patient Match Technology was used (Visionaire^®^, Smith & Nephew, Memphis, TN). Individual cutting blocks made of nylon were produced based on three dimensional models from MRI and standing long-leg radiographs [16].

### Radiological analysis

Anterior–posterior (a.p.) and lateral radiographs of the knee joint as well as a.p. radiographs of the entire leg were aquired under full weight-bearing. The leg was in neutral rotation with the patella facing straight forward, the fibular head covered by the tibia for one third and correct projection of the trochanter minor and ankle [17–20]. Geometrical angles and axes were measured using the software mediCad. The mTFA was defined as the angle between the mechanical tibial axis and the mechanical femoral axis. The mechanical axis of the femur was measured from the center of the femoral head to the center of a line drawn from the medial to the lateral femoral epicondyles. The mechanical axis of the tibia was measured from the ankle talus center to the center of a line drawn from the medial to the lateral edge of the bony resection surface of the proximal tibia plateau [18, 21–23]. Positive values were set for varus and negative values for valgus alignment. The medial proximal tibia angle (MPTA) was defined as the medial angle between the mechanical axis of the tibia and the bony resection surface of the proximal tibia [17, 23].

### Statistical analysis

Statistics were calculated using “R” and the software RStudio^©^ (RStudio, Inc., Boston, USA). Data were analyzed concerning normal/nonnormal distribution using histograms *QQ*-plots and mean/median. Analysis of variance (ANOVA) was used to assess differences between the four groups, pairwise testing was done with sequential Bonferroni correction (Holm’s method). Results with normal distribution are presented as means with standard deviation, results with nonnormal distribution are presented as medians with interquartile ranges. Logistic regression was performed to demonstrate the odd’s ratio (OR) of rendering an outlier for each method. Logistic regression results are presented as OR and 95% confidence interval (CI). A *p* value < 0.05 was considered statistically significant. Correlations were displayed with scatter plots and calculated using Pearsons’s correlation coefficient.

## Results

**Fig. 1:**
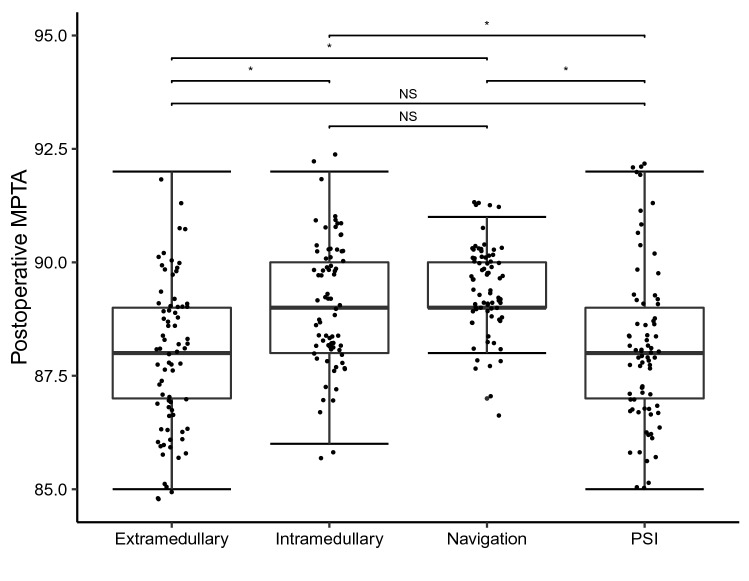
Dot plot displaying the postoperative medial proximal tibia angle (MPTA) for each method separately. Each dot represents one patient. Boxplots displaying the median with interquartile ranges of the postoperative MPTA for each method. Asterisks indicate significant differences between indicated groups

The accuracy of frontal tibial positioning was determined through measurement of the postoperative MPTA. Using ANOVA we could show that the preoperative MPTA was not significantly different distributed between all groups, hence demonstrating that deformities were equally randomized between groups. The postoperative MPTA, however, was significantly different distributed between all groups. The mean postoperative MPTA closest to the neutral alignment of 90° was in the navigation group (Table 1). Post-hoc analysis showed that navigation and the intramedullary technique were each significantly more precise in reconstructing a neutrally aligned postoperative MPTA compared to PSI and the extramedullary technique (Fig. 1). Significant differences between the pre- and postoperative MPTA could only be found in the navigation and intramedullary group. We did not find any significant differences in postoperative MPTA between the navigation and intramedullary technique. In the extramedullary and PSI group, pre- and postoperative MTPA did not differ significantly. Subsequently, reconstruction of the MPTA using the extramedullary or PSI technique was deemed insufficient in this cohort.

**Table 1:** Baseline characteristics and results of all four groups

	Extramedullary	Intramedullary	Navigation	PSI
*n*	75	75	75	75
Age	67.5 (7.3)	66.9 (7.8)	67.2 (7.6)	66.9 (7.2)
Sex = m (%)	32 (42.7)	33 (44.0)	29 (38.7)	29 (38.7)
BMI	29.5 (2.7)	28.8 (2.9)	29.2 (2.8)	29.2 (2.7)
Weight	83.7 (11.1)	91.8 (72.2)	84.9 (10.6)	86.01 (10.3)
ASA	2.0 [2.0, 3.0]	2.00 [2.0, 3.0]	2.0 [2.0, 3.0]	2.00 [2.0, 3.0]
mTFA preoperative	4.2 (4.5)	4.21 (4.2)	4.3 (4.3)	4.60 (4.6)
mTFA postoperative	1.95 (1.8)	0.93 (1.7)	0.7 (1.4)	1.71 (2.1)
mTFA outlier = yes (%)	10 (13.3)	5 (6.7)	3 (4.0)	14 (18.7)
MPTA preoperative	87.85 (1.4)	88.00 (1.6)	88.2 (1.6)	88.00 (1.4)
MPTA postoperative	87.92 (1.6)	89.08 (1.4)	89.4 (0.9)	88.11 (1.7)
Slope preoperative	6.32 (1.1)	6.41 (1.1)	6.5 (1.1)	6.63 (1.0)
Slope postoperative	6.03 (1.2)	4.63 (1.4)	5.0 (2.0)	3.43 (1.2)

Values are given as mean with standard deviation (curved brackets) or as median with interquartile ranges (square brackets)

*BMI* Body mass index, *ASA* American Society for Anesthesiologists Score, *mTFA *mechanical tibiofemoral angle, *MPTA* medial proximal tibia angle

**Fig. 2:**
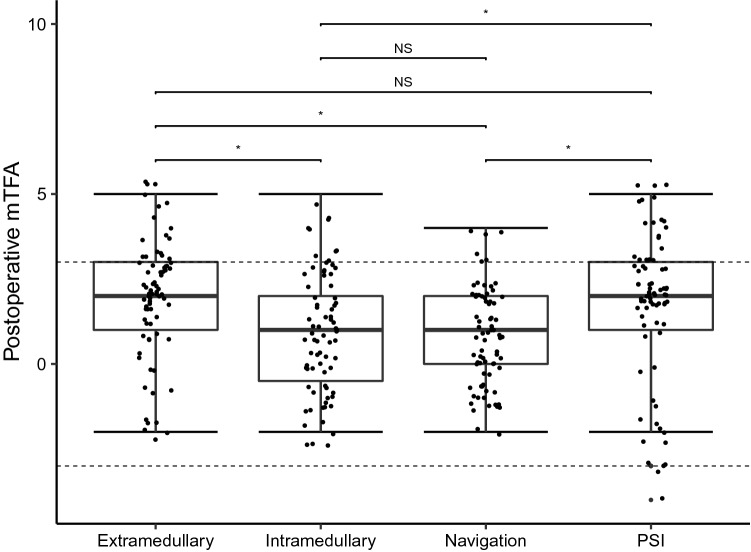
Dot plot displaying the postoperative mechanical tibiofemoral angle (mTFA) for each method separately. Each dot represents one patient. The dashed lines indicate the cutoff for outliers (mTFA > 3° from the neutral axis). Boxplots displaying the median with interquartile ranges of the postoperative mTFA for each method. Asterisks indicate significant differences between indicated groups

ANOVA testing equally showed that the preoperative mTFA was not significantly different distributed between all groups, demonstrating that malalignments were equally randomized between groups, as well. The postoperative mTFA was significantly different distributed between all groups. The mean postoperative mTFA closest to the neutral axis could be found in the navigation group (Table 1). Post-hoc analysis reveled that navigation and the intramedullary technique were each significantly more precise in reconstructing a neutral mTFA compared to PSI and the extramedullary technique (Fig. 2). We could not find significant differences between navigation and the intramedullary technique concerning postoperative mTFA (Fig. 2). Further analysis demonstrated that the postoperative mTFA was significantly improved compared to the preoperative mTFA in each technique.

PSI produced the highest rate of outliers (mTFA > 3° from the neutral axis) (18.7%) followed by the extramedullary technique (13.3%). Navigation and intramedullary positioning rendered relatively low rates of outliers of merely 4.0% and 6.7%, respectively (Table 1; Fig. 2). Logistic regression could demonstrate that the odd’s ratio for producing outliers was highest for the PSI method. PSI OR = 5.5 (1.5–20.1 95% CI), *p* < 0.05; extramedullary positioning OR = 3.7 (1.0–14.0 95% CI), *p* > 0.05; intramedullary positioning OR = 1.7 (0.4–7.4 95% CI), *p* > 0.05; navigation OR = 0.04, *p* < 0.05. Outliers are depicted for each method separately in Fig. 2.

**Fig. 3a, b:**
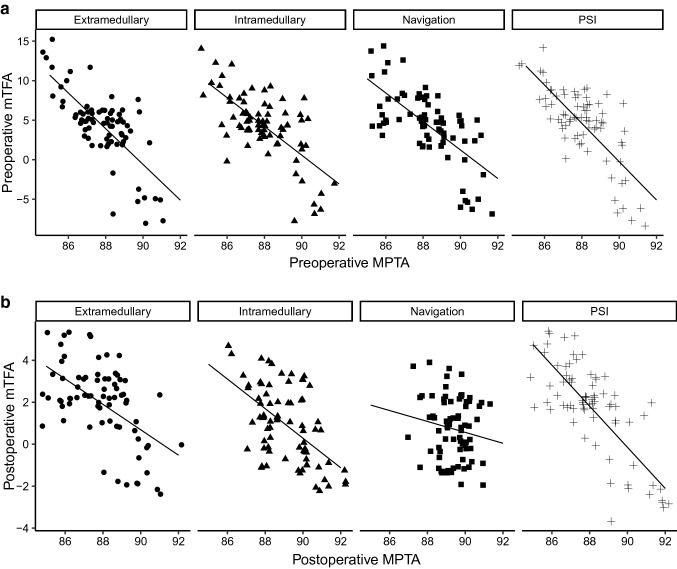
Correlation analysis between the medial proximal tibial angle (MPTA) and the mechanical tibio-femoral axis (mTFA) both (**a)** preoperatively and (**b)** postoperatively with subsets for each technique separately. **a** Pre-operative: extramedullary *R* = − 0.72, *p* < 0.05; intramedullary *R* = − 0.69, *p* < 0.05; PSI *R* = − 0.75, *p* < 0.05; navigation *R* = − 0.67, *p* < 0.05. **b** Postoperative: extramedullary *R* = − 0.55, *p* < 0.05; intramedullary *R* = −0.56, *p* < 0.05; PSI *R* = − 0.77, *p* < 0.05; navigation *R* = − 0.16, *p* > 0.05. Pearson’s correlation testing

Correlation analysis could demonstrate that the preoperative MPTA significantly correlated with the preoperative mTFA in all groups, indicating that there was an association between the tibial alignment and the mechanical axis of the entire lower limb (extramedullary *R* = −0.72, *p* < 0.05; intramedullary *R* = −0.69, *p* < 0.05; PSI *R* = −0.75, *p* < 0.05; navigation *R* = −0.67, *p* < 0.05) (Fig. 3a). In analyzing the association between the MPTA and mTFA postoperatively we could observe that both parameters significantly correlated in all groups but the navigation technique (extramedullary *R* = −0.55, *p* < 0.05; intramedullary *R* = −0.56, *p* < 0.05; PSI *R* = −0.77, *p* < 0.05; navigation *R* = −0.16, *p* > 0.05) (Fig. 3b). The decreasing correlation between the postoperative MPTA and mTFA in the navigation group reflect our above mentioned observations of the most accurate reconstruction of the MPTA with the navigation technique. These results underline the importance of precise tibial alignment.

Baseline characteristics did not differ between groups and are depicted in Table 1.

## Discussion

Despite being the most frequently used method for tibial instrumentation in primary TKA worldwide, a significant amount of outliers with the tibial component has been evaluated in our study for the extramedullary technique. The accuracy of the tibial cut is particularly important when performing a tibia-first technique. In the current study, the method of tibial instrumentation significantly influenced the accuracy of MPTA and mTFA. The innovative PSI technique failed to demonstrate superior accuracy with a high rate of outliers for MPTA and mTFA.

Different factors have been identified to impede the accuracy of extramedullary orientation like obesity or drapings covering bony structures. Operative techniques have been reported to improve the accuracy in tibial extramedullary instrumentation [6]. Several methods to facilitate orientation in assistance with anatomical landmarks have been reported. A high precision of 98% for alignment has been described after identifying the center of the talus [24]. Another study advised palpating the anterior tibial crest which is within 3° of the tibial mechanical axis [13]. Furthermore, the distance between the extramedullary rod and the bone should be kept as short as possible to avoid errors in the tibial cut [25].

Less outliers regarding MPTA and MA were observed with the intramedullary technique, a method with a long historical tradition. Similar to our analysis, a study including 103 TKAs without severe bone deformities detected less outliers and a more accurate coronal alignment with the intramedullary compared to the extramedullary technique [26]. Intramedullary instrumentation was shown to be safe and accurate in patients with no or minor deformities. Patients with previous surgery of the affected knee and significant deformities have been excluded in our study.

The results of our study demonstrated a significantly higher accuracy in tibial positioning of the intramedullary compared to the extramedullary technique. There is no evidence in the literature favoring one of these two conventional methods over the other [6, 27]. In fact, outlier rates have been reported as high as 37% and 33% for the extra- and intramedullary technique, respectively [6]. In comparison, our results demonstrated outlier rates of merely 10% and 5% for these two techniques. Rahm et al. reported 31.5% outliers in the extramedullary group, 34.6% in the navigational group and 24.4% in the PSI group [28]. The preoperative mTFA of the referenced studies was markedly higher (14–20°) than the preoperative mTFA in our study (4° across all groups, Table 1) [8, 28, 29]. The low rate of outliers in our study could be conditioned by the differences in the baseline characteristics, and by exclusion of excessive preoperative deformities with a low variation of mTFA in our study. In comparison, other studies which demonstrated high rates of outliers did not report the preoperative mTFA [6, 30]. The comparability is, therefore, restricted. Furthermore, in several studies, different surgeons had performed the operations [28]. In this study, one single senior surgeon performed all operations, improving the consistency of the results [31].

Within the last years, two innovative concepts for alignment of TKA have been established which both attempt to overcome the limitations of the conventional techniques. Navigation underwent an enormous progress as it started about 30 years ago as computer-assisted surgery and developed into imageless navigation [15]. The navigation system used in our study was based on optical landmarks. Results after navigation are discussed controversely. Some authors have shown that navigation was superior for restoring alignment compared to conventional methods in literature [15, 32]. Others could not demonstrate significant differences concerning the postoperative alignment of the components nor the mechanical axis and outliers [33]. A recently published multicenter randomized control trial was the first to also show better functional outcome 2 years after navigated TKA compared to conventional instrumentation [34]. Previous generation navigation systems could not demonstrate superior long time outcome before [35]. Acquisition of the technique, surgery time and financial aspects are further important factors that have to be considered in navigated TKA.

Patient-Specific-Instrumentation has been thought to overcome the unsatisfied patient after TKA due to consideration of the individual anatomy. Promising results for PSI have been initially described in the literature. Within the following years, PSI failed to prove its superiority regarding alignment and clinical outcome compared to conventional methods [36–40]. The results of our study were in line with these observations, similar results concerning the percentage of mTFA outliers had been reported for PSI before [41]. Interestingly, an improvement in the accuracy of the femoral component in PSI has been reported in a meta analysis [16]. Coincident, the risk for tibial malalignment was found to be increased, which is in accordance with the findings of our study, although we did not measure the femoral positioning [16, 42]. Yamamura et al. reported that in PSI, CT-based 3D-measurements demonstrated vast differences between the preoperative planning of the implants and the actual position of the implants after the operation, particularly for the tibial component [43]. But other authors described an improved tibial component rotation using PSI [44]. Different factors can potentially influence tibial component position in PSI and render different study results, such as the preoperative planning and imaging modalities, manufacturing process and material characteristics of patient-specific cutting guides as well as their intraoperative positioning. In PSI, financial aspects and decreased operation times have to be considered, too.

Our study has several limitations. The follow-up was restricted to three postoperative months. But as previously reported, no further change in mTFA alignment after TKA could be observed after three months [22]. Nevertheless, clinical relevant findings may emerge in the long run. The current study was limited to radiological analysis and did not evaluate clinical parameters. The clinical relevance is given by the analysis of outliers. Furthermore, we only analyzed the frontal tibial positioning and did not measure the sagittal tibial and femoral alignment. Because MPTA did not significantly change in the extramedullary and PSI group despite observing significant changes in both groups concerning mTFA, femoral component alignment may have an important impact on whole leg alignment. On the other hand, femoral positioning is highly dependent on tibial alignment in the tibia-first technique, which was used in this study. Nevertheless, a future study with the same setup should analyze the accuracy of the femoral alignment and its impact on whole-leg alignment in all four techniques. Moreover, two different TKA systems were used in this study. However, when performing the study, there was no single TKA system for all four techniques available. Therefore, to minimize a possible systematic error, two TKA systems of the same producer were employed.

## Conclusions

In this study, four different techniques of tibial resection in TKA performed by one single senior surgeon were directly compared to each other prospectively for the first time. Accuracy of tibial component position was dependent on the technique of tibial instrumentation. Overall alignment in TKA was significantly influenced by the tibial component positioning. Optimal alignment can only be achieved with the highest precision techniques. As innovative techniques fail to outperform conventional methods, surgeons need to verify and reevaluate their preferred method of tibial instrumentation consistently during TKA.
